# A Comparison between Dietary Consumption Status and Healthy Dietary Pattern among Adults Aged 55 and Older in China

**DOI:** 10.3390/nu14132778

**Published:** 2022-07-05

**Authors:** Siting Zhang, Liusen Wang, Xiaofang Jia, Jiguo Zhang, Hongru Jiang, Weiyi Li, Feifei Huang, Huijun Wang, Bing Zhang, Gangqiang Ding, Zhihong Wang

**Affiliations:** Key Laboratory of Trace Elements Nutrition, National Institute for Nutrition and Health, Chinese Center for Disease Control and Prevention, National Health Commission, Beijing 100050, China; zsiting1015@163.com (S.Z.); wangls@ninh.chinacdc.cn (L.W.); jiaxf@ninh.chinacdc.cn (X.J.); zhangjg@ninh.chinacdc.cn (J.Z.); jianghr@ninh.chinacdc.cn (H.J.); liwy@ninh.chinacdc.cn (W.L.); huangff@ninh.chinacdc.cn (F.H.); wanghj@ninh.chinacdc.cn (H.W.); zhangbing@chinacdc.cn (B.Z.); dinggq@chinacdc.cn (G.D.)

**Keywords:** dietary intake, recommended intake, middle-aged and elderly, China

## Abstract

The nutrition and health of middle-aged and elderly people is crucial to the long-term development of a country. The present study aimed to analyze the dietary consumption status in Chinese adults by using baseline and follow-up data from the community-based Cohort Study on Nervous System Diseases between 2018–2020 and selecting those aged 55 and older (*n* = 23,296). Dividing 65 food items into 17 subgroups on the basis of a valid semi-quantitative food frequency questionnaire, we analyze the consumption amount and consumption rate of foods in relation to wave and sociodemographic factors by employing the Wilcoxon rank sum test, Kruskal–Wallis analysis, the Chi-squared test, and the Cochran–Armitage trend test and evaluate food intake status using the Chinese Dietary Guidelines Recommendations (2022). Compared to 2018, the median daily intake of livestock meat, poultry, and eggs increased in 2020 (*p* < 0.05), while the median daily intake of wheat, other cereals, tubers, legumes, fruits, and fish and seafood decreased (*p* < 0.05). The proportion of subjects with excessive intake of grain, livestock and poultry, and eggs was 46.3%, 36.6%, and 26.6%, respectively, while the proportion of subjects with insufficient intake of whole grains and mixed beans, tubers, legumes, dairy products, fruits, vegetables, and fish and seafood were 98.4%, 80.3%, 74.0%, 94.6%, 94.3%, 75.8%, and 86.5%, respectively, and more than 50% of subjects were non-consumers of dairy products, nuts, and whole grains and mixed beans. In conclusion, the problem of unhealthy dietary structure is prominent among adults aged 55 and older in China; insufficient or excessive intakes of various types of foods are common; and excessive consumption of edible oil and salt remains a serious problem.

## 1. Introduction

China has experienced dramatic aging of its population and a health transition over the past few decades [1,2]. As of 2020, the population aged 60 years and above was 264 million, accounting for 18.7% of the country′s total, 190 million of whom were aged 65 years and above [3]. It is predicted that there will be 402 million (28%) Chinese aged 60 and older by 2040 [4]. Environment, lifestyle and behavioral factors, and care or treatment are essential to address and prepare for the challenges of aging and staying healthy, such as chronic disorders and physical frailty [5]. Among these challenges, diet and nutrition play an important role in promoting healthy aging [6,7,8].

With economic growth and urbanization in China comes a nutritional transition mainly manifested as changes in eating and cooking behaviors [9,10], food preference and dietary knowledge [11], and Westernization of dietary patterns [12]. From 1991 to 2015 in China, it is noteworthy that the population converted to consume more animal-source foods, processed foods, dairy products, and fruits [12]; over a similar time frame, the consumption of grains, especially coarse grains, tubers, and vegetables declined, and fruit intake remained low [13]. A 2015 study suggested that dietary structure among Chinese adults aged 60 and older was not rational and estimated the proportions of the population with a lower intake of vegetables, fruits, soybeans, and dairy products than the recommended amounts, which were roughly up to 65%, 95%, 80%, and 99%, respectively [14]. The available evidence suggests that high intake of fruits, vegetables, fish, (whole) grains and legumes, and potatoes are associated with healthy longevity and better cardiometabolic and cognitive health, whereas dietary patterns rich in red meat and sugar-rich foods are inverse associations [6,7]. In addition, a population-based study prospectively confirmed that adherence to various healthy dietary patterns at midlife was associated with higher likelihood of healthy aging at later life, such as the alternative Mediterranean Diet (aMED, OR_Q4VSQ1_ = 1.52, 95% CI: 1.31–1.77, *p*-trend < 0.001) or the Dietary Approaches to Stop Hypertension Diet (DASH, OR_Q4VSQ1_ = 1.53, 95% CI: 1.35–1.73, *p*-trend < 0.001) [15].

In response to the transition in dietary structure and nutritional status of the Chinese in recent years, the Chinese Dietary Guidelines Recommendations (2022) was officially published in April 2022. This document is committed to guiding residents to choose food scientifically and apportion it rationally, thus preventing chronic diseases and improving health quality [16]. Compared to the 2016 edition [17], the latest guidelines emphasize the intake of whole grains, two servings of seafood per week, 300–500 g of dairy products per day, and a further limit of salt intake to <5 g/day [16]. “Oriental healthy dietary patterns” is a highlight of the document that encourages healthy dietary behaviors and balanced dietary patterns [16]. However, to our best knowledge, few studies have used the latest recommendations of the China dietary guidelines to evaluate dietary nutritional status and analyze new dietary consumption status among the middle-aged and elderly.

In the context of an aging society with nutritional transition in China, we must comprehensively understand the dietary consumption status and identify nutritional problems among the middle-aged and elderly before we can recommend interventions and provide specific nutrition education. Therefore, the present study aimed to analyze the dietary consumption status of Chinese adults aged 55 and older using baseline and follow-up data from the community-based Cohort Study on Nervous System Diseases (CCSNSD) carried out in 2018 and 2020, respectively, as well as with a focus on food intake compared with the Chinese Dietary Guidelines Recommendations (2022) [16].

## 2. Materials and Methods

### 2.1. Study Population

The data in the present study are derived from the CCSNSD, a longitudinal study established by the National Institute for Nutrition and Health, Chinese Center for Disease Control and Prevention in 2018, which focused on potential factors associated with risks of nervous diseases, including epilepsy, Alzheimer’s disease and Parkinson’s disease, of which the first follow-up survey was finished in 2020. A multistage, stratified random sampling process was used to draw participants without such diseases in Hebei, Zhejiang, Shaanxi, and Hunan provinces. The survey design has been reported in detail elsewhere [18]. A total of 13,443 participants aged 55 and older who met the inclusion and exclusion criteria were recruited at baseline, and 12,840 participants completed the follow-up survey, with a follow-up rate of 95.5%. The protocol of this study was reviewed and approved by the Institutional Review Board of the National Institute for Nutrition and Health (No. 2017020, 6 November 2017). In addition, written informed consent was obtained from all the participants before the survey.

The present study used the baseline and follow-up survey data from the CCSNSD. Subjects aged 55 years and older with complete data of sociodemographic characteristics and dietary surveys were selected. We excluded subjects with implausible energy intake (<400 or >6000 kcal/day, *n* = 652 responses) and those with incomplete dietary (*n* = 2054 responses) or sociodemographic data (*n* = 281 responses). Finally, the total number of participants involved in the analysis were 12,167 and 11,129 in 2018 and 2020, respectively.

### 2.2. Assessment of Food Consumption

Dietary information was assessed by a validated semi-quantitative food frequency questionnaire (FFQ) covering 65 food items categorized in 17 major food groups and items in this study [19]: rice, wheat, other cereals, tubers, legumes, whole grains and mixed beans, vegetables, fruits, livestock meat, poultry, organ and processed meat, fish and seafood, eggs, dairy products, nuts, snacks, and water and beverages (Table S1). The sections of the questionnaire dealing with nutrient supplements were not included. The participants were asked about their dietary habits during the previous 12 months, including the frequency of each food item (daily, weekly, monthly, annually, or never) and the amount consumed each time. Of the dairy products and legumes, the amount of the corresponding products was converted into the amount of liquid milk or dry beans according to their protein ratios per 100 g of edible parts, respectively. As for common edible oil and condiments, we recorded the average monthly household consumption and the number of family members who usually ate at home, then calculated their average daily intake of edible oil and salt (g/day). According to the 2009 and 2018 China Food Composition [20,21], the total daily energy intake for each person was estimated by summing the energy contribution from each food item and from the edible oil and condiments.

For each item, if the participant reported “never”, then his/her food intake or consumption frequency was equal to zero. For consumers, food intake was calculated according to the reported average intake amount and frequency of each food item. Dietary consumption status was evaluated from the following three aspects: (1) consumption rate: the ratio of the number of subjects who consumed the food to the total number of subjects; (2) consumption amount: based on the above-mentioned food intake and expressed as daily intake (g/day or mL/day); (3) comparison with the recommendations: the estimated consumption amount of the food group was compared with that reported in the Chinese Dietary Reference Guidelines (2022) [16] (Table S2), and the proportions of subjects with food intake below, within, or above the recommended range were evaluated, respectively, indicating “insufficient”, “adequate”, or “excessive”. In addition, the subjects who did not consume particular foods were defined as “non-consumers”.

### 2.3. Measurement of Sociodemographic Characteristics

Health workers who received two rounds of training by national or provincial experts and passed a qualification test employed questionnaires to collect sociodemographic information for each subject, including gender (male or female), age (55–64, 65–74, ≥75 years), educational level (illiterate, primary school and below, middle school, and high school and above), residential region (rural or urban), and monthly household income per capita (<1000, 1000–3999, ≥4000 [Chinese yuan RMB]).

### 2.4. Statistical Analysis

Data analysis was performed by SAS version 9.4 (SAS Institute, Inc., Cary, NC, USA). Continuous variables were presented as median and interquartile (IQR), where categorical variables were expressed as *n* (%). Due to the non-normal distribution, the non-parametric Wilcoxon rank sum test or Kruskal–Wallis H test were conducted to examine differences in the consumption amount of foods by wave and sociodemographic factors. If the difference was significant among more than two groups, an additional multiple comparison of the Dwass-Steel-Critchlow-Fligner (DSCF) method was performed. The proportion of food consumption rates by wave and sociodemographic factors was analyzed using Chi-squared test, and the Cochran–Armitage trend test was employed to further explore trends in the subgroups of participants according to age group, educational level, and monthly household income per capita. According to the foods recommended as “plenty”, “moderate”, or “limit” by the dietary guidelines (2022), we further analyzed the distribution of severely insufficient or excessive intake of major foods. All statistical tests were two-tailed and considered significant with a value of *p* < 0.05.

## 3. Results

### 3.1. Baseline Characteristics of the Study Population

Among the sample of 12,167 adults aged 55 and older included in the study at baseline, 82.8% were 55–74 years old, about 56.6% were females, 51.9% were from rural areas, 43.6% had a primary school education or below, and 78.3% had a monthly household income per capita of more than RMB 1000, respectively.

### 3.2. Differences in Dietary Consumption Status between the Years 2018 and 2020

The median intake of livestock meat, poultry, and eggs was 28.6, 6.7, and 30.0 g/day in 2020, respectively, increasing by 0.5, 1.7, and 4.2 g/day as compared to 2018 *(p* < 0.05), while the median daily intakes of wheat, other cereals, tubers, legumes, fruits, and fish and seafood were 57.1, 7.5, 16.7, 6.5, 33.2, and 6.7 g in 2020, respectively, decreasing by 17.9, 3.2, 4.4, 1.9, 9.0, and 0.5 g as compared to 2018 (*p* < 0.05). The median daily intake of dairy products was 0.0 g, and the percentage of the population that consumed it dropped from 41.3% in 2018 to 39.0% in 2020 (*p* < 0.05). Similarly, the median intake and consumption rate of nuts dropped significantly (0.7 vs. 0.0 g/day; 56.6% vs. 46.6%; *p* < 0.05). Overall, participants tended to consume rice, wheat, tubers, legumes, vegetables, fruits, livestock meat, and eggs, while more than 50% of them reported not consuming whole grains and mixed beans, organ and processed meat, dairy products, nuts, and snacks (Table 1).

### 3.3. Sociodemographic Disparity in Dietary Consumption Status in 2020 

Gender, age, residence area, educational level, and monthly household income per capita significantly influenced the distribution of the dietary consumption amount (Table 2). In females, the daily intake of rice, wheat, and livestock meat was lower and that of fruits was higher than in males. Participants aged ≥75 years had a lower intake of rice, wheat, legumes, vegetables, fruits, livestock meat, poultry, and fish and seafood than those aged 55–64 and 65–74 years (*p* < 0.05). Urban residents tended to eat more rice, tubers, legumes, fruits, poultry, and fish and seafood and less wheat, livestock meat, edible oil, and salt than rural residents (*p* < 0.05). The daily fruits, legumes, poultry, eggs, fish and seafood, and water and beverage intake presented increasing trends associated with increased educational level and monthly household income per capita (*p* < 0.05), while the daily edible oil and salt intake presented the adverse trends (*p* < 0.05). Table S3 presents the consumption rates of foods by sociodemographic characteristics.

### 3.4. Proportion of Subjects with Insufficient, Adequate, or Excessive Food Intake Compared to Chinese Dietary Guidelines Recommendations in 2020 

**Figure 1:**
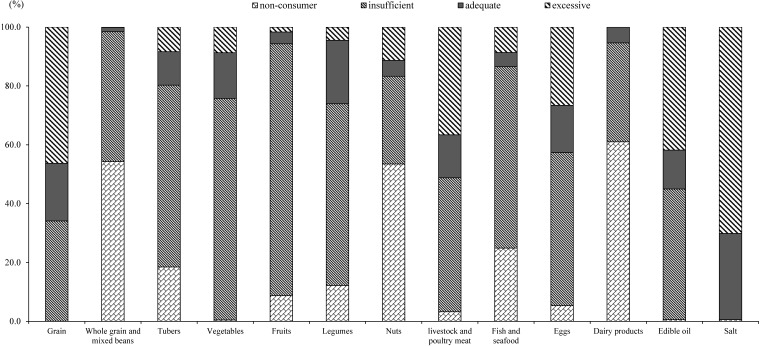
Comparisons of daily food intakes and recommendations among Chinese adults aged 55 years and above in four provinces in 2020.

As shown in Figure 1, of all participants, the proportion of subjects with excessive grain intake was 46.3%, while the proportions of subjects with insufficient intake of whole grains and mixed beans, tubers, and legumes were 44.1%, 61.8%, and 61.8%, respectively, and 54.3%, 18.5%, and 12.2% were non-consumers. As regards vegetables and fruits, 75.3% and 85.6% of the population had insufficient intake, 0.5% and 8.7% were non-consumers, and only 15.6% and 4.0% ate adequately, respectively. The proportion of subjects consuming excessive amounts of livestock and poultry meat and eggs were 36.6% and 26.6%, respectively, while 61.6% of subjects consumed fish and seafood insufficiently, with 24.9% being non-consumers. Only 5.4% of subjects had an adequate intake of nuts and dairy products. 41.8% and 70.1% of the subjects consumed edible oil and salt excessively, respectively.

**Figure 2:**
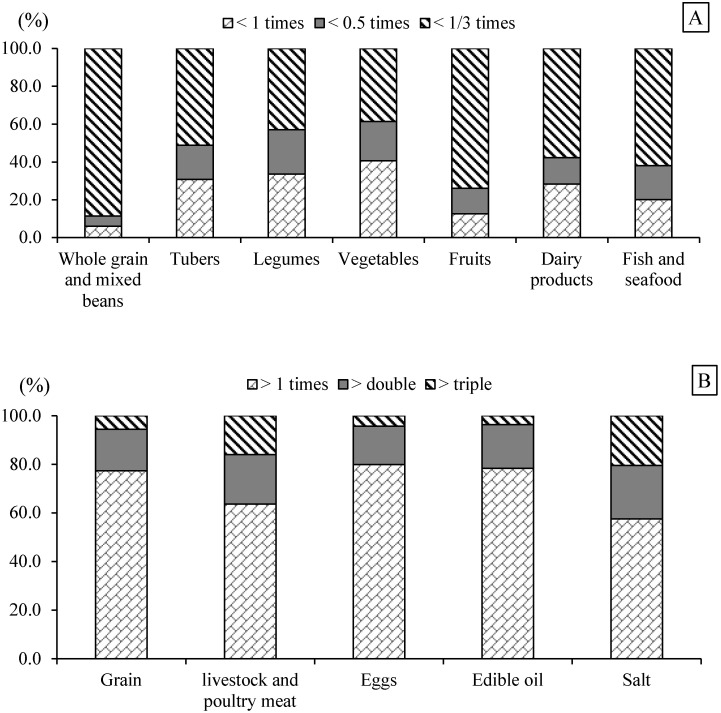
Distributions of insufficient (**A**) or excessive (**B**) intake of major foods among Chinese adults aged 55 years and above in four provinces in 2020.

Among the consumers with insufficient or excessive intake, the percentages having an intake less than a third of the recommendation for whole grains and mixed beans, tubers, legumes, vegetables, fruits, dairy products, and fish and seafood were 88.6%, 51.2%, 42.9%, 38.6%, 74.0%, 57.7%, and 62.0%, respectively. The percentages having an intake more than one time the recommendation for grain, livestock and poultry meat, eggs, edible oil, and salt were 77.4%, 63.6%, 79.9%, 78.4%, 57.5%, respectively. 20.4% and 21.9% of the subjects consumed more than twice the recommended quantity of livestock and poultry meat and salt, respectively, and 16.0% and 20.6% consumed more than three times the recommended amount (Figure 2).

## 4. Discussion

The present study, which used a validated semi-quantitative FFQ, observed dietary status and its differences with the Chinese Dietary Guidelines Recommendations (2022) among adults aged 55 years above in four Chinese provinces. Compared with 2018, the median daily intake of livestock meat, poultry, and eggs increased, whereas the inverse directions were found for the intake of wheat, other cereals, tubers, legumes, fruits, and fish and seafood in 2020, and more than 50% of the participants were reported not to consume whole grains and mixed beans, dairy products, and nuts during the previous year. Moreover, an unhealthy dietary structure was prominent, mainly manifested as high proportions of excessive intake of grains (46.3%) and insufficient intake of tubers (80.3%: 61.8% of insufficient +18.5% of non-consumer) and whole grains and mixed beans (98.4%: 44.1% + 54.3%), and excessive intake of livestock and poultry meat (36.6%) and eggs (26.6%) and insufficient intake of fish and seafood (86.5%: 61.6% + 24.9%), along with severely insufficient daily intake of legumes (74.0%: 61.8% + 12.2%), dairy products (94.6%: 33.6% + 61.0%), fruits (94.3: 85.6% + 8.7%), and vegetables (75.8%: 75.3% + 0.5%). In addition, the percentages with excessive consumption of edible oil and salt were 41.8% and 70.1%, respectively.

The China National Nutritional and Health Survey reported that the average dietary intake of grains, tubers, and vegetables declined from 1982 to 2017 [22,23]; in agreement with these findings, the intake of dietary fiber which mainly comes from grains, vegetables, and fruits has been in a progressive decline over the past decades [24], with the consumption of refined grains increasing. Numerous studies reported that higher coarse grain consumption, including whole grains, was associated with lower blood pressure and lower risk of hypertension, diabetes, and ischemic stroke among Chinese adults [25,26,27]. In the present study, the improper composition of staple foods in 2020 was also found among adults aged 55 and older, which showed excessive intake of grains but seriously inadequate intake of whole grains, mixed beans, and tubers. Higher consumption of whole grains was significantly associated with a lower risk of type 2 diabetes (HR_Q5VSQ1_ = 0.71, 95% CI: 0.67–0.74, *p*-trend < 0.001) [28], cardiovascular disease (per 90 g/day increasing: RR = 0.78, 95% CI: 0.73–0.85), and all-cause mortality (RR = 0.83, 95% CI: 0.77–0.90) [29], so the above-mentioned improper consumption might pose potential risks for long-term health [30]. The consumption of legumes and its products, which are rich in high-quality protein and phytochemicals, remains low in our study. Previous prospective cohort studies showed that higher intake of soy isoflavones (HR_Q5VSQ1_ = 0.87, 95% CI: 0.81–0.94, *p*-trend = 0.008) and tofu (HR = 0.82, 95% CI: 0.70–0.95, *p*-trend = 0.005) was associated with a moderately lower risk of developing coronary heart disease [31]. Several meta-analyses also indicated that women with high dietary soy foods intake may experience a significant reduction in the risk of breast cancer [32] and postmenopausal osteoporosis [33]. Moreover, in contrast to Europe and North America where daily vegetable and fruit consumption increased from 2002 to 2010 [34], levels of vegetable and fruit intake in China were so insufficient as partially to cause more than 60% of the population to be at risk of vitamin C inadequacy [35].

A high percentage of energy from dietary fat has been a prominent factor in unhealthy diet in Chinese adults. The results from the China Health and Nutrition Survey in 2015 showed that the proportion of the elderly whose percentage of energy from dietary fat exceed 30% was 61.7% [36]; this might be closely related to a dietary structure featuring excessive intake of livestock and poultry meat but insufficient intake of aquatic products along with excessive consumption of edible oil. Between 1991 and 2011, the meat consumption patterns of Chinese adults were characterized by a predominant intake of pork, suboptimal intakes of seafood, and an increased proportion of adults having excessive intakes of red meat and poultry [37]. In 2015, livestock meat intake constituted 86.9% of the total meat intake, and pork intake constituted 86.5% of the livestock meat intake among the elderly [38]. High dietary fat intake from meat and edible oil, especially livestock meat rich in saturated fatty acids, can have adverse health effects, such as obesity and cardiovascular system disorders [39,40], but moderate intake of meat including seafood and edible oil remains an important source of polyunsaturated fatty acids and micronutrients. Furthermore, it is not negligible that excessive salt consumption contributes to the high incidence of chronic diseases in China [16]; 70.1% of the subjects in our study consume more than the recommended amount, and nearly half of them consume twice the recommended amount. Although the daily intake of dairy products has increased slightly over the past three decades, the overall levels were still low [41]; only 5.4% of the participants met the Chinese dietary recommendations in 2020 in the present study. 

Taken together, grains and livestock meat are the most prominent elements in the diet of the middle-aged and elderly in China, while the consumption of whole grains, vegetables, fruits, legumes, and dairy products, which is recommended in plentiful amounts, remains low. This is likely due to traditional diet habits developed in youth as well as to food sources and economic factors [9,12]. As the latest edition of the Chinese dietary guidelines shows, the dietary patterns in the Zhejiang, Shanghai, and Jiangsu provinces in China have been regarded as representative of “Oriental healthy dietary patterns,” which are characterized by food diversity, less oil and salt, plentiful intake of fresh vegetables and fruits, relatively high intake of fish and seafoods, and low intake of pork, along with high intake of dairy and beans [16]. Numerous studies confirmed that healthy dietary patterns were associated with lower risk of cardiovascular diseases, hypertension, cancer, and type 2 diabetes [42]. However, the Global Burden of Disease Study (2017) estimated that unhealthy diet was the main cause of many diseases and deaths in China, and the number of deaths attributed to poor diet quality was 3.1 million [2]. Thus, it is evident that population-specific nutrition education and intervention programs are urgently needed to improve dietary consumption status among Chinese adults in accordance with the dietary guidelines.

Our study has several limitations. First, because the participants came from only four of China’s provinces, one should be cautious about generalizing the findings to other regions of the country. Second, dietary consumption status over the past 12 months was estimated by an FFQ as self-reported measurements, which may lead to recall biases and potential underestimation. Third, the average distribution of daily edible oil and salt intake might lead to an inaccurate assessment of each person’s actual intake. However, this is a large study evaluating the food intake status of Chinses adults compared with the latest published dietary guidelines in 2022, and a population-based design for CCSNSD can reduce the selection bias. Moreover, comprehensive evaluation of the nutrition and health of the subjects by trained health workers can increase the internal validity of the results.

## 5. Conclusions

In conclusion, unhealthy dietary structure is a prominent problem among adults aged 55 and older in China. It is mainly manifested in high proportions of the population with excessive intake of grains and of livestock and poultry meat and insufficient intake of tubers, whole grains and mixed beans, fish and seafood, daily dairy products, and fruits and vegetables; a particularly serious problem is the excessive consumption of edible oil and salt. Population-specific nutrition education and intervention programs are urgently needed to improve dietary consumption status in accordance with the 2022 Dietary Guidelines.

## Figures and Tables

**Table 1:** Dietary Consumption Status of Chinese Adults Aged 55 Years and Above in Four Provinces in CCSNSD 2018–2020.

Foods	Consumption Amount (g/day) ^a^		*p*-Value	Consumption Rate ^b^		*p*-Value
	2018	2020		2018	2020	
Rice	120.0 (42.9, 225.0)	120.0 (28.6, 240.0)	0.987	11,787 (96.9)	10,672 (95.9)	<0.001
Wheat	75.0 (21.4, 150.0)	57.1 (14.3, 120.0)	<0.001	11,138 (91.5)	9711 (87.3)	<0.001
Other cereals	10.7 (1.0, 28.6)	7.5 (0.0, 28.0)	<0.001	9386 (77.1)	8076 (72.6)	<0.001
Tubers	21.1 (6.7, 44.9)	16.7 (4.2, 40.0)	<0.001	10,475 (86.1)	9073 (81.5)	<0.001
Whole grains and mixed beans	0.7 (0.0, 5.7)	0.0 (0.0, 3.0)	<0.001	6888 (56.6)	5087 (45.7)	<0.001
Vegetables	169.9 (84.3, 298.0)	168.5 (85.7, 294.5)	0.854	12,058 (99.1)	11,079 (99.6)	<0.001
Fruits	42.2 (16.7, 87.4)	33.2 (12.7, 72.0)	<0.001	11,523 (94.7)	10,161 (91.3)	<0.001
Legumes	8.4 (3.0, 17.5)	6.5 (2.6, 15.5)	<0.001	10,963 (90.1)	9774 (87.8)	<0.001
Livestock meat	28.1 (10.5, 57.1)	28.6 (12.9, 57.1)	0.002	11,578 (95.2)	10,499 (94.3)	0.005
Poultry	5.0 (1.1, 14.3)	6.7 (1.7, 14.3)	<0.001	9893 (81.3)	9224 (82.9)	0.002
Organ and processed meat	0.0 (0.0, 2.7)	0.0 (0.0, 1.4)	<0.001	4682 (38.5)	3778 (34.0)	<0.001
Fish and seafood	7.2 (0.8, 28.6)	6.7 (0.1, 21.4)	<0.001	9465 (77.8)	8359 (75.1)	<0.001
Eggs	25.8 (12.9, 57.1)	30.0 (14.3, 51.7)	<0.001	11,247 (92.4)	10,539 (94.7)	<0.001
Dairy products	0.0 (0.0, 60.6)	0.0 (0.0, 63.3)	0.051	5029 (41.3)	4338 (39.0)	<0.001
Nuts	0.7 (0.0, 5.7)	0.0 (0.0, 3.3)	<0.001	6888 (56.6)	5180 (46.6)	<0.001
Snacks	0.1 (0.0, 7.1)	0.0 (0.0, 5.3)	<0.001	6119 (50.3)	4831 (43.4)	<0.001
Water and beverage *	813.3 (500.0, 1300.0)	800.0 (480.0, 1220.0)	<0.001	11,700 (96.2)	10,672 (95.9)	0.295
Edible oil	27.8 (17.5, 40.0)	26.2 (16.7, 41.7)	<0.001	12,162 (100.0)	11,067 (99.4)	<0.001
Salt	5.5 (3.6, 7.5)	6.7 (4.2, 10.8)	<0.001	12,157 (99.9)	11,068 (99.5)	<0.001

^a^ Values are expressed as Median (Q1,Q3) and examined using Wilcoxon rank sum test. ^b^ Values are expressed as *n* (%) and examined using Chi-Square Test. * The unit of water and beverage in consumption amount is ml/day.

**Table 2:** Consumption amount of foods by characteristics among Chinese adults aged 55 years and above in four provinces in CCSNSD 2020 ^1^.

Characteristics	Rice	Wheat	Other Cereals	Tubers	Vegetables	Fruits	Legumes	Livestock Meat	Poultry	Fish and Seafood	Eggs	Water and Beverage	Edible Oil	Salt
Gender														
Male	120.3	60.0	7.4	16.7	164.3	30.0	6.4	30.9	6.7	6.7	30.1	900.0	26.7	6.7
Female	120.0	51.4	7.5	16.8	171.1	35.2	6.6	28.6	6.7	6.7	30.0	800.0	25.1	6.7
*p*-Value	0.007	<0.001	0.861	0.932	0.145	<0.001	0.711	<0.001	0.120	0.670	0.429	<0.001	0.007	0.399
Age group (years) ^2^														
55–64	140.0 ^a^	57.1 ^a^	7.1	17.0	171.4 ^a^	34.7 ^a^	6.6	31.3 ^a^	6.7 ^a^	6.7 ^a^	30.0	900.0 ^a^	26.7 ^a^	6.7
65–74	120.0 ^a^	57.1	8.0	17.1	174.6 ^a^	34.3 ^a^	6.7 ^a^	29.0 ^a^	6.7 ^b^	6.7 ^a^	30.2	800.0 ^b^	26.7	6.7
≥75	120.0 ^b^	51.4 ^b^	7.1	15.7	153.6 ^b^	28.6 ^b^	6.4 ^b^	27.3 ^b^	4.7 ^c^	5.6 ^b^	29.8	800.0 ^b^	25.0 ^b^	6.7
*p*-Value	0.006	0.012	0.049	0.062	<0.001	<0.001	0.041	<0.001	<0.001	<0.001	0.594	<0.001	0.026	0.073
Residential area														
Rural	100.0	75.0	8.2	14.8	166.9	30.1	6.4	30.0	5.7	4.0	29.7	800.0	26.7	6.8
Urban	150.0	50.0	7.1	18.0	169.3	36.4	7.7	28.6	6.7	8.8	30.4	880.0	25.3	6.7
*p*-Value	<0.001	<0.001	0.034	<0.001	0.762	<0.001	<0.001	<0.001	<0.001	<0.001	0.038	0.124	<0.001	<0.001
Education level ^2^														
Illiteracy	100.0 ^a^	60.0 ^a^	7.6 ^bc^	16.0 ^a^	154.1 ^a^	26.8 ^a^	6.4 ^a^	23.1 ^a^	5.0 ^a^	3.3 ^a^	28.6 ^a^	720.3 ^a^	30.8 ^c^	7.2 ^c^
Primary school and below	150.0 ^b^	50.0 ^b^	6.7 ^b^	15.3 ^a^	170.9 ^b^	30.7 ^b^	6.4 ^b^	29.1 ^b^	5.8 ^b^	6.7 ^b^	28.6 ^a^	800.0 ^b^	25.8 ^b^	6.7 ^b^
Middle school	100.0 ^a^	71.4 ^a^	10.0 ^a^	18.0 ^b^	182.3 ^c^	39.8 ^c^	7.2 ^c^	30.2 ^bc^	6.7 ^b^	6.7 ^b^	34.3 ^b^	1000.0 ^c^	25.0 ^ab^	6.7 ^ab^
High school and above	140.0 ^b^	57.1 ^b^	8.6 ^ac^	18.7	157.3 ^ab^	45.7 ^d^	8.8 ^d^	33.5 ^c^	7.1 ^c^	10.5 ^c^	42.9 ^c^	1000.0 ^c^	23.3 ^a^	6.5 ^a^
*p*-Value	<0.001	<0.001	<0.001	<0.001	<0.001	<0.001	<0.001	<0.001	<0.001	<0.001	<0.001	<0.001	<0.001	<0.001
Monthly household income per capital (RMB) ^2^														
<1000	42.9 ^a^	100.0 ^a^	13.3 ^a^	18.0 ^a^	155.8 ^a^	26.7 ^a^	6.4 ^a^	21.4 ^a^	4.0 ^a^	1.6 ^a^	40.5 ^a^	800.0	30.0 ^a^	7.7 ^a^
1000~3999	142.9 ^b^	57.1 ^b^	6.7 ^b^	16.3	178.8 ^b^	34.3 ^b^	6.9 ^b^	30.7 ^b^	6.7 ^b^	6.7 ^b^	29.7 ^b^	810.0	25.0 ^b^	6.3 ^b^
≥4000	200.0 ^c^	34.3 ^c^	6.7 ^c^	17.0 ^b^	151.4 ^a^	36.8 ^b^	6.9 ^b^	30.7 ^b^	7.1 ^c^	14.3 ^c^	28.6 ^c^	807.1	25.0 ^c^	7.2 ^c^
*p*-Value	<0.001	<0.001	<0.001	0.073	<0.001	<0.001	<0.001	<0.001	<0.001	<0.001	<0.001	0.043	<0.001	<0.001

^1^ Values are expressed as median and examined using Wilcoxon rank sum test or Kruskal–Wallis analysis, and the units are g/day except water and beverage measured by mL/day; the median daily intakes of whole grains and mixed beans, organ and processed meat, dairy products, nuts, and snacks were almost 0.0 g by sociodemographic factors, so their data were not shown. ^2^ Subgroups with different superscript letters were significantly different by multiple comparison of DSCF (Dwass-Steel-Critchlow-Fligner).

## Data Availability

No additional data are available.

## Supplementary Materials

The following supporting information can be downloaded at: https://www.mdpi.com/article/10.3390/nu14132778/s1, Table S1: Food grouping used in the dietary consumption analysis; Table S2: Recommendations of foods derived from Chinese Dietary Guidelines (2022); Table S3: Consumption rates of foods by characteristics among Chinese adults aged 55 years and above in four provinces in CCSNSD 2020.
