# Supplementary material for: A Comparison between Dietary Consumption Status and Healthy Dietary Pattern among Adults Aged 55 and Older in China
Source: Nutrients. 2022 Jul 5;14(13):2778. doi: 10.3390/nu14132778 (PMC9268783; doi:10.3390/nu14132778)
Supplement: Supplementary file 1 [file nutrients-14-02778-s001.zip › nutrients-1781067-supplementary.pdf]

Table S1. Food grouping used in the dietary consumption analysis

food or food groups	Foods included in the group
Rice	Cooked white rice, cooked rice with assorted mixtures, and rice-noodle
Wheat	Non-fried noodles (bread, steamed buns, noodles, dumplings)
Other cereals	Coarse cereals (corn, barley, sorghum, rye), fried noodles, and instant noodles
Tubers	Sweet potato, potato, and taro
Legumes	Soybean, and products
Whole grains and mixed beans	Whole-wheat bread, buckwheat, mung beans, and red beans
Vegetables	legume vegetables, tomatoes, peppers, melon vegetables, green leafy vegetables, cabbage and other leafy vegetables, cruciferous vegetables, mushroom, alga, dried and fermented vegetables
Fruits	Orange fruits, melon fruits, berry fruits, all other fresh fruits, and dried or canned fruit
Livestock meat	Pork, beef, lamb, and mutton
Poultry	Chicken, duck, goose, pigeon, and quail
Organ and processed meat	Organ meat, sausage, ham sausage, and luncheon meat
Fish and seafood	Fish, shrimp, crab, and shellfish
Eggs	Fresh egg, salted egg, and preserved egg
Dairy products	Liquid milk, milk powder, yogurt, and cheese
Nuts	Peanuts, melon seeds, pumpkin seeds, and watermelon seeds
Snacks	Cakes, cookies, pastries, bread, biscuit, chocolate, chips and other fried snacks
Water and beverage	No added water or soda, kola, other sugared beverages, strong tea, coffee, and fruit juice

Table S2. Recommendations of foods derived from Chinese Dietary Guidelines (2022)

Food or food groups	Recommendations		Unit
	55-64 years	>=65 years	
Grain	200~300	200~250	g/day
Whole grains and mixed beans	50~150	50~150	g/day
Tubers	50~100	50~75	g/day
Legumes	105~175	105	g/week
Vegetables	300~500	300~450	g/day
Fruits	200~350	200~300	g/day
Animal Food	120~200	120~150	g/day
livestock and poultry meat	280~525	280~350	g/week
Fish and seafood	280~525	280~350	g/week
Eggs	280~350	280~350	g/week
Dairy products	300	300	g/day
Nuts	50~70	50~70	g/week
Edible oil	25~30	25~30	g/day
Salt	<5	<5	g/day

Table S3. Consumption rates of foods by characteristics among Chinese adults aged 55 years and above in four provinces in CCSNSD 2020.[1]

Characteristics	Rice	Wheat	Other cereals	Tubers	Whole grains and mixed beans	Vegetables	Fruits	Legumes	Livestock meat	Poultry	Organ and processed meat	Fish and seafood	Eggs	Dairy products	Nuts	Snacks	Water and beverage	Edible oil	Salt
Gender																			
Male	95.7	88.5	72.2	80.5	43.4	99.5	90.0	87.9	95.0	83.1	36.1	74.7	94.4	35.9	44.8	41.5	95.8	99.6	99.6
Female	96.1	86.3	72.9	82.3	47.5	99.6	92.3	87.8	93.9	82.7	32.3	75.4	94.9	41.3	47.9	44.9	96.0	99.4	99.4
p-Value	0.318	<0.001	0.390	0.013	<0.001	0.517	<0.001	0.864	0.014	0.543	<0.001	0.375	0.283	<0.001	<0.001	<0.001	0.539	0.204	0.243
Age group (years)																			
55-64	96.2	87.9	71.3	81.3	45.2	99.6	92.0	87.9	95.2	84.9	35.7	76.5	94.6	37.7	50.4	42.7	96.0	99.6	99.7
65-74	96.4	86.9	73.2	81.6	45.9	99.5	91.9	88.3	94.5	83.3	34.0	75.5	94.6	38.8	47.3	43.1	95.8	99.3	99.3
>=75	94.4	87.2	73.2	81.7	46.0	99.5	89.0	86.7	92.9	79.1	31.4	72.3	95.1	41.3	39.4	45.1	95.9	99.4	99.4
p-Value	0.001	0.893	0.072	0.689	0.467	0.585	<0.001	0.201	<0.001	<0.001	<0.001	<0.001	0.367	0.006	<0.001	0.072	0.751	0.163	0.164
Residential area																			
Rural	94.5	87.3	70.4	78.0	43.5	99.2	88.0	87.0	94.2	78.3	27.4	66.9	94.2	32.0	45.4	41.8	94.3	99.9	99.9
Urban	97.2	87.3	74.6	84.8	47.8	99.9	94.4	88.7	94.4	87.2	40.1	82.9	95.2	45.6	47.6	44.9	97.4	99.0	99.0
p-Value	<0.001	0.967	<0.001	<0.001	<0.001	<0.001	<0.001	0.006	0.643	<0.001	<0.001	<0.001	0.014	<0.001	0.019	<0.001	<0.001	<0.001	<0.001
Education level																			
Illiteracy	93.8	87.2	69.3	76.3	39.0	99.5	86.1	84.7	92.9	79.6	29.3	66.8	93.5	32.1	42.0	42.3	96.3	99.4	99.3
Primary school and below	96.4	85.1	72.6	83.9	46.0	99.6	91.6	87.7	94.0	83.9	36.4	76.2	94.2	35.3	47.8	46.2	95.4	99.2	99.3
Middle school	95.4	89.9	73.9	83.1	48.0	99.4	92.7	89.7	95.0	83.1	31.2	75.1	94.9	42.6	46.2	40.0	95.1	99.6	99.6
High school and above	98.2	89.1	74.7	80.0	50.6	99.8	95.6	89.7	96.2	84.4	37.7	83.2	97.2	52.6	49.7	42.5	97.9	99.8	99.8
p-Value	<0.001	<0.001	<0.001	0.007	<0.001	0.498	<0.001	<0.001	<0.001	<0.001	<0.001	<0.001	<0.001	<0.001	<0.001	0.067	0.061	0.015	0.011
Monthly household income per capital (RMB)																			
<1000	89.8	91.8	78.7	77.3	39.2	99.3	84.4	85.8	89.4	74.6	27.6	60.5	94.7	32.9	38.6	39.1	94.7	98.6	98.7
1000~3999	96.7	88.1	70.8	82.4	46.7	99.5	92.1	88.3	95.0	84.1	34.5	75.3	94.7	38.8	47.3	45.0	95.7	99.6	99.6
>=4000	99.4	81.1	71.5	83.2	49.1	100.0	95.7	88.4	97.3	87.4	38.5	87.9	94.9	45.0	52.0	43.5	97.5	99.8	99.8
p-Value	<0.001	<0.001	<0.001	<0.001	<0.001	<0.001	<0.001	0.006	<0.001	<0.001	<0.001	<0.001	0.738	<0.001	<0.001	0.003	<0.001	<0.001	<0.001

[1] Values are expressed as % and examined using Chi-Square test or Cochran-Armitage trend test.
